# Evaluating the safety and efficacy of *Lacticaseibacillus paracasei* TISTR 2593 as a therapeutic probiotic for obesity prevention

**DOI:** 10.3389/fmicb.2025.1501395

**Published:** 2025-04-02

**Authors:** Jaruwan Sitdhipol, Kanidta Niwasabutra, Neungnut Chaiyawan, Kamonsri Nuankham, Thanaphol Thanagornyothin, Somboon Tanasupawat, Wasaporn Preteseille Chanput, Pongsathon Phapugrangkul, Chaivarakun Chaipanya, Sukanya Phuengjayaem, Saranporn Poothong, Engkarat Kingkaew

**Affiliations:** ^1^Biodiversity Research Centre, Research and Development Group for Bio-Industries, Thailand Institute of Scientific and Technological Research, Pathum Thani, Thailand; ^2^Faculty of Pharmaceutical Sciences, Department of Biochemistry and Microbiology, Chulalongkorn University, Bangkok, Thailand; ^3^Faculty of Agro Industry, Department of Food Science and Technology, Kasetsart University, Bangkok, Thailand; ^4^Faculty of Science, Department of Microbiology, King Mongkut’s University of Technology Thonburi, Bangkok, Thailand; ^5^Faculty of Veterinary Science, Department of Animal Husbandry, Chulalongkorn University, Bangkok, Thailand; ^6^Department of Biology, School of Science, King Mongkut’s Institute of Technology Ladkrabang, Bangkok, Thailand

**Keywords:** obesity, antiadipogenic, probiotics, *Lacticaseibacillus paracasei*, whole-genome sequencing, probiogenomic analysis

## Abstract

Several recent studies have reported the potential of probiotics in reducing body weight and fat mass and improving glucose and lipid metabolism. Therefore, probiotic administration is considered an alternative approach for treating obesity. The objective of this study was to evaluate the probiotic properties and antiadipogenic potential of the strain TISTR 2593. Through whole-genome sequence analysis, the strain TISTR 2593 was identified as *Lacticaseibacillus paracasei*. *L. paracasei* TISTR 2593 exhibited *γ*-hemolytic activity (nonhemolysis) and demonstrated susceptibility to antibiotics, indicating that it is generally safe for consumption. Additionally, this strain displayed desirable probiotic properties, including tolerance to artificial gastric juice and bile salts, adhesion to Caco-2 cells, and the ability to inhibit pathogens. Furthermore, *L. paracasei* TISTR 2593 exhibited cholesterol-reducing capability and demonstrated antiadipogenic activity. In 3T3-L1 adipocytes, treatment with 10% (w/v) heated *L. paracasei* TISTR 2593 cells resulted in an approximately 50% reduction in lipid accumulation, similar to the positive control (quercetin). Moreover, *L. paracasei* TISTR 2593 heat-killed cells dose-dependently decreased the expression levels of CCAAT/enhancer-binding protein-*α* and peroxisome proliferator-activated receptor-*γ*, two vital transcription factors involved in the early stage of adipocyte differentiation. These findings suggest that *L. paracasei* TISTR 2593 possesses probiotic and functional properties, including antiadipogenic activity, supporting its potential as a therapeutic probiotic supplement for preventing obesity. Overall, the results of this study indicate that *L. paracasei* TISTR 2593 exhibits promising probiotic characteristics and beneficial effects on adipogenesis modulation, reinforcing its potential as a therapeutic option in obesity prevention.

## Introduction

Obesity is defined as the excessive accumulation of fat, which is a common risk factor for noncommunicable diseases (NCDs), such as type 2 diabetes, cardiovascular disease, hypertension, stroke, various forms of cancer, and mental health issues. Moreover, obesity has been linked to increased morbidity and mortality in patients with COVID-19 (Boutari and Mantzoros, 2022; Fruh, 2017). In March 2022, the World Health Organization (WHO) projected that more than 1 billion people worldwide would be obese. By 2025, an estimated 167 million individuals, including both adults and children, are expected to experience health complications associated with being overweight or obese. On World Obesity Day 2022, the WHO called on countries to strengthen their efforts in addressing and reversing this preventable and foreseeable health crisis (WHO, 2022).

In recent years, multiple studies have reported the potential of probiotics to suppress obesity (León Aguilera et al., 2022). Probiotics possess species-specific or strain-specific beneficial effects and could be utilized in humans if they possess the required properties to confer beneficial effects, such as resistance to acids and bile, proliferation on and adherence to intestinal epithelial cells (Ouwehand et al., 2002). The antiobesity effects of probiotics have been attributed to their ability to alter the gut microbiota, remodel energy metabolism, modify the expression of genes associated with thermogenesis, glucose metabolism, and lipid metabolism, and impact parasympathetic nerve activity (León Aguilera et al., 2022).

Several probiotic lactic acid bacteria (LAB) have emerged as promising strategies for the prevention and treatment of obesity in the future (Shen et al., 2022; Tsai et al., 2014). Multiple studies have indicated that LAB exert an inhibitory effect on lipid accumulation during adipogenesis by downregulating the key activators PPAR-γ and C/EBP-α. In a recent study, Kim et al. (2020) reported that treatment with cell-free extracts (CFEs) of *Lactobacillus fermentum* MG4231 and MG4244 reduced lipid accumulation and intracellular triglyceride production in 3T3-L1 adipocytes by more than 50%, which was mediated by the downregulation of FAS and aP2 resulting from the inhibition of PPAR-γ and C/EBP-α gene expression. Guha et al. (2021) demonstrated that *Lactobacillus delbruckei* subsp. *bulgaricus* and *Streptococcus thermophilus* could reduce cellular lipids and downregulate the expression of mammalian adipogenesis marker genes in terminally differentiated 3T3-L1 cells. Similarly, Han et al. (2022) reported that *Lactobacillus plantarum* KU15117 exhibited antiadipogenic activity in 3T3-L1 cells and suppressed the expression of adipocyte-specific genes and proteins.

The Biodiversity Research Centre of Thailand Institute of Scientific and Technological Research (TISTR), specifically the Innovative Center for Production of Industrially Used Microorganisms, operates a probiotics bank with the objective of isolating, collecting, researching, and conserving local probiotics for their industrial use in foods and functional products. The present study aimed to identify a potential new probiotic from our bank for its ability to prevent obesity. Among the candidates, the potential *L. paracasei* TISTR 2593 was selected for evaluation because of its potential antiadipogenic activities.

## Materials and methods

### Isolation and identification of strain TISTR 2593

The strain TISTR 2593 was isolated from breast milk two days after childbirth and subsequently preserved during the TISTR culture collection. Initial identification of the strain TISTR 2593 was conducted through Gram-staining, catalase testing, and assessment of acid production on MRS agar supplemented with CaCO_3_. Clear zones were observed around colonies on MRS medium supplemented with 1% (w/v) CaCO_3_, suggesting the likely classification of this strain as lactic acid bacteria (LAB). For ongoing research, the strain was maintained at −80°C in 15% (v/v) glycerol until use.

Genomic DNA extraction followed the protocol outlined in Wilson (2001). Whole-genome sequencing utilized hybrid technologies, such as the Oxford Nanopore Technologies (ONT) with the Rapid Sequencing Kit and MinIONTM device, alongside the Illumina platform with the NextSeq® 500 high-output kit v2 (300 cycles) for improved accuracy. Trim Galore (Galaxy Version 0.6.3) was employed to remove adaptors and low-quality reads to ensure data quality, followed by processing with the Unicycler genome assembly program (Galaxy Version 0.4.8.0).

16S rRNA gene sequences were extracted via ConEST16S (Lee et al., 2017), and 16S rRNA gene sequences were extracted via ConEST16S (Yoon et al., 2017) to determine sequence similarity. The average nucleotide identity (ANI) and digital DNA–DNA hybridization (dDDH) values were calculated via the JSpeciesWS web server tool (Richter and Rosselló-Móra, 2009; Richter et al., 2016) and the Genome-to-Genome Distance Calculator (GGDC 2.1) with the BLAST+ method and formula 2 (Kim et al., 2014). Additionally, average amino acid identity (AAI) was analyzed via the Kostats laboratory (Rodriguez-R and Konstantinidis, 2014). Finally, a circular genomic map was generated via Proksee (Grant et al., 2023).

### Functional genome analysis of strain TISTR 2593

The whole genome was annotated via the DFAST server (Tanizawa et al., 2018), Rapid Annotation Server Technology (RAST) (Aziz et al., 2008), PATRIC (Davis et al., 2020), and the NCBI Prokaryotic Genome Annotation Pipeline (PGAP) (Tatusova et al., 2016). Antibiotic resistance genes were determined via the Comprehensive Antibiotic Resistance Database (CARD; https://card.mcmaster.ca) (Alcock et al., 2020) and the ResFinder web-based tool (Bortolaia et al., 2020). Pathogenicity was predicted via the PathogenFinder web-based tool (Cosentino et al., 2013), and plasmids were detected via PlasmidFinder (Carattoli et al., 2014). The Kyoto Encyclopedia of Genes and Genomes (KEGG) database (https://www.kegg.jp) (Kanehisa et al., 2016) was used to search for pathways and genes.

### Hemolysis activity

Strain TISTR 2593 was streaked onto 5% (v/v) sheep blood agar and then incubated anaerobically at 37°C for 24 h. Following the incubation period, the plates were examined for the presence of a clear zone (*β*-hemolysis), a greenish zone (*α*-hemolysis), or no zone (*γ*-hemolysis, nonhemolytic) around the colonies.

### Antibiotic susceptibility

The antibiotic susceptibility assay was conducted against a broad spectrum of clinically important antibiotics via the disc diffusion method as outlined by the Clinical and Laboratory Standards Institute (Humphries et al., 2021). Seven antibiotics, including ampicillin (10 μg), chloramphenicol (30 μg), vancomycin (30 μg), tetracycline (30 μg), erythromycin (15 μg), kanamycin (30 μg), and clindamycin (2 μg), were utilized. The cell concentration of each bacterial culture was adjusted to match McFarland No. 1 standards and then applied onto Mueller–Hinton (MH) agar (Merck, Darmstadt, Germany) via a sterile cotton swab, followed by a 10-min incubation at room temperature. The antibiotic discs were subsequently aseptically placed onto the agar. *Staphylococcus aureus* ATCC 6538 and *Escherichia coli* ATCC 8379 were employed as positive controls. The agar plates were then incubated under optimal conditions for bacterial growth, and the inhibition zones were measured and compared to the breakpoint values specified by Humphries et al. (2021).

### Antimicrobial activity

Antimicrobial activity was assessed via the agar-well dffusion assay method outlined by Ennahar et al. (2000). Six pathogenic bacteria—*S. aureus* ATCC 6538, *Escherichia coli* ATCC 8379, *Salmonella* Typhimurium TISTR 292, *Salmonella enteritidis* DMST 15676, *Listeria monocytogenes* DMST 13820, and *Helicobacter pylori* PT14—served as indicator strains. Following cultivation for 18–24 h under their optimal growth conditions, each indicator strain was suspended in 0.85% (w/v) NaCl, and the cell density was adjusted to the McFarland No. 1 standard (10^8^ CFU/mL). These suspensions were then overlaid onto nutrient agar (NA) via a cotton swab, and sections of NA were aseptically removed via Corkborer No. 3. The LAB culture mixture was subsequently centrifuged overnight for 10 min at 10,000 × g, and 70 μL of the resulting cell-free supernatant was added to each well. After 24 h of incubation at 37°C, the diameter of the inhibition or clear zone was measured. Sterile MRS broth was used as negative control.

### Acid and bile salt tolerance

Simulated gastrointestinal fluid (SGI) was prepared according to the modified method of Hyronimus et al. (2000). In brief, 0.1% (w/v) pepsin was dissolved in MRS broth containing 0.05% (w/v) L-cysteine, and the pH was adjusted to 2 with 1 M HCl and 1 M NaOH. The solution was then sterile-filtered through a 0.2 μm membrane (Life Sciences, Ann Arbor, MI, USA) and either used immediately or refrigerated until needed (for no longer than 24 h). Bile salt tolerance was assessed as per the procedure outlined by Gilliland et al. (1985). Bile salts at a concentration of 0.3% (w/v) were dissolved in MRS broth supplemented with 0.05% (w/v) L-cysteine at pH 8 and sterilized via a liquid cycle for 15 min. One milliliter of overnight culture (OD600 = 1.0) in MRS broth was subsequently inoculated into 9.0 milliliters of SGI (pH 2) and 0.3% (w/v) bile salt solution (pH 8). The resulting mixtures were immediately evaluated for the viability of candidate probiotics via the standard pour plate method and then incubated at 37°C for 180 min. The viability of the remaining probiotics was assessed via the aforementioned methods, and the survival rate was calculated as the logarithmic value of colony-forming units per milliliter (Log10CFU/mL) according to the following formula:


$$\mathrm{Percentage of surviving cells}\;\left(\%\right)=\left(\log_{10}N_{1}/\log_{10}N_{0}\right)\times 100$$

where N_1_ is the average base 10 logarithm of viable cells (Log10CFU/mL) after incubation for 180 min.

N_0_ is the average base 10 logarithm of the number of viable cells (Log10CFU/mL) at the initial incubation time (0 min).

### Adhesion assay

The ability of strain TISTR 2593 to adhere to human epithelial cells was tested following the protocol outlined by Kingkaew et al. (2022a). First, human colorectal adenocarcinoma cells (Caco-2) (ATCC, HTB-37) were cultured in Dulbecco’s modified Eagle’s medium (DMEM) and seeded into a 24-well cell culture plate at a density of 2 × 10^5^ cells/well. The plate was then incubated at 37°C in a 5% CO_2_ humidified incubator for 14 days. Viable cells of strain TISTR 2593 were harvested by centrifugation (8,000 × g, 10 min, 4°C) and washed twice with sterile PBS (pH 7.2). The cell density was adjusted to 1 × 10^9^ CFU/mL with DMEM. Next, 1 mL of the strain TISTR 2593 cell suspension was added to each well and incubated at 37°C in a 5% CO_2_ atmosphere for 1 h. Following incubation, the Caco-2 cells were washed three times with sterile PBS to remove nonadherent TISTR 2593 cells. The Caco-2 cells from the monolayers were detached with 1% (v/v) Triton X-100. The suspension containing TISTR 2593 cells was enumerated via 10-fold serial dilution and spread onto MRS agar. After incubation for 48 h at 37°C, the adhesion ability was determined via the following formula:


$$\mathrm{Percentage of adhesion ()}=\left(N_{1}/N_{0}\right)\times 100$$

where N_1_ represents the average base 10 logarithm of adherent bacterial cells (CFU/mL) after incubation.

N_0_ represents the average base 10 logarithm of non-adherent bacterial cells (CFU/mL) added before incubation as a control.

### Cholesterol assimilation assay

The strain TISTR 2593 was cultivated in MRS broth containing 100 μg/L cholesterol-PEG 600 and incubated at 37°C for 24–48 h under anaerobic conditions. The strain TISTR 2593 cells were subsequently removed via centrifugation at 4,000 × g for 10 min at 4°C, and the supernatants were separated for cholesterol assimilation determination. Cholesterol levels were determined via a modification of the method described by Rudel and Morris (1973). Briefly, 100 μL of the supernatant sample was added to 200 μL of 95% (v/v) ethanol and 100 μL of 33% (w/v) potassium hydroxide. This mixture was vortexed for 1 min and then heated in a water bath at 60°C for 10 min. After cooling, 500 μL of hexane and a 1 mL aliquot of distilled water were added and thoroughly mixed. The mixture was then allowed to stand at room temperature to allow phase separation. A 100 μL aliquot of the hexane layer was transferred to a clean 96-well plate and evaporated under a flow of nitrogen gas. Next, 200 μL of freshly prepared O-phthalaldehyde (0.5 mg O-phthalaldehyde per ml of acetic acid) was added to each well of the 96-well plate containing the mixture. After the addition of 50 μL of concentrated sulfuric acid and standing for an additional 15 min, the absorbance at 550 nm was read against the reagent blank. The absorbance values were then compared with those obtained with cholesterol standards.


A=(C-B/C)×100

where A is the cholesterol assimilation (as a percentage),

B is the cholesterol level in the test sample.

C is the cholesterol level in the control (uninoculated MRS broth containing cholesterol-PEG 600 (100 ug/L)).

### Cell line and cell culture

3T3-L1 preadipocyte cells, which are mouse embryonic fibroblasts, were maintained in Dulbecco’s modified Eagle’s medium (DMEM) supplemented with 4.5 g/L glucose, 10% (v/v) fetal bovine serum, and 1% (v/v) penicillin/streptomycin at 37°C with 5% CO_2_ in a humidified incubator. The cells were subcultured every 5–6 days when they reached 70–80% confluence.

### Cytotoxicity

Heat-inactivated cells of the strain TISTR 2593 were tested for cytotoxicity via the 3-(4,5-dimethylthiazol-2-yl)-2,5-diphenyltetrazolium bromide (MTT) method. Adipocyte 3T3-L1 cells were seeded in 96-well cell culture plates at a density of 1× 10^4^ cells/well and incubated with heat-inactivated TISTR 2593 cells at concentrations up to 10% (v/v) for 24 h at 37°C with 5% CO_2_ in a humidified incubator. After incubation, the cells in the wells were washed twice with phosphate buffer solution, and 20 μL of MTT staining solution (5 mg/mL in phosphate buffer solution) was added to each well. The well plate was then incubated at 37°C for 3 h. Following incubation, 100 μL of dimethyl sulfoxide was added to each well to dissolve the formazan crystals, and the absorbance was read at 570 nm via an microplate reader. The percentage of cell viability was calculated via microplate reader the following formula:


$$\mathrm{Percentage of cell viability ()}=\left(\frac{\mathrm{Absorbance}\;\mathrm{of}\;\mathrm{sample}}{\mathrm{Absorbance}\;\mathrm{of}\;\mathrm{control}}\right)\times 100$$

### Lipid accumulation

To investigate the effect of strain TISTR 2593 on lipid accumulation, 3T3-L1 preadipocytes were differentiated via the methods outlined by Nasution et al. (2020) and Udomwasinakun et al. (2022). Heat-inactivated cells of strain TISTR 2593 at concentrations of 2.5, 5, and 10% (v/v) were added to the differentiation medium cocktails I and II, as described by Udomwasinakun et al. (2022). The control condition comprised 10% (w/v) peptone water, matching the highest volume used for heat-inactivated cells of strain TISTR 2593. Quercetin at 30 μg/mL served as the positive control, given its well-documented antiadipogenic activity (Udomwasinakun et al., 2022). Mature 3T3-L1 adipocytes on day 9 were harvested by fixing them in 10% (v/v) formalin at room temperature for 1 h before lipid accumulation was analyzed via the Oil-red-o solution 0.50% (v/v) in isopropanol staining assay, following the protocol outlined by Nasution et al. (2020). The oil droplet size and number were observed under an inverted microscope (Olympus IX73, Japan). The stained cells were decolorized with isopropanol before the absorbance was measured at 490 nm with a microplate reader. The percentage of lipid accumulation was expressed relative to that of the control (peptone water).

### Expression of the C/EBP-*α* and PPAR-*γ* genes

The RNA of 3T3-L1 cells was extracted via a commercial GeneJET RNA purification kit (Thermo Fisher Scientific, USA) and purified with DNase I amplification grade (Thermo Fisher Scientific, USA). First-strand cDNA was synthesized from 200 uL of RNA via the commercial RevertAid First Strand cDNA Synthesis Kit (Thermo Fisher Scientific, USA) following the manufacturer’s protocol. After reverse transcription, 500 uL of cDNA was subjected to real-time PCR amplification with iTaqTM Universal SYBR® Green Supermix (Bio-Rad, USA) with the following primers: *β*-actin (F: 5’-GGCTGTATTCCCCTCCATCG-3′, R: 5’-CCAGTTGGTAACAATGCCATGT-3′), PPAR-γ (F: 5’-GGAAGACCACTCGCATTCCTT-3′, R: 5’-GTAATCAGCAACCATTGGGTCA-3′), and C/EBP-α (F: 5’-GCGGGAACGCAACAACATC-3′, R: 5’-GTCACTGGTCAACTCCAGCAC-3′). Real-time PCR (Bio-Rad CFX96TM Real-Time System, USA) was employed for amplification. The extracted cDNA was amplified with a thermocycler. The cycle threshold (Ct) was normalized to that of β-actin as a reference gene and reported relative to the control (peptone water).

### Statistical analysis

All the experiments were performed in triplicate, and the results are presented as the mean ± standard deviation. The results were subjected to analysis of variance (one-way ANOVA) via SPSS software (version 22.0). Significant differences among the means (*p* < 0.05) were evaluated via Duncan’s new multiple range test (DMRT).

## Results and discussion

### Isolation and characterization of strain TISTR 2593

The strain TISTR 2593 is a Gram-positive, rod-shaped bacterium that tests negative for catalase. The complete genome of strain TISTR 2593 contains one copy of the 16S rRNA gene, and the 16S rRNA gene sequence of strain TISTR 2593 has been deposited in GenBank under accession number LC759806. The 16S rRNA gene sequences were highly similar to those of several *Lacticaseibacillus* species, i.e., 100% to *L. paracasei* subsp. *tolerans* JCM 1171^T^, 99.93% to *L. paracasei* subsp. *paracasei* ATCC 25302^T^, 99.35% to *L. zeae* ATCC 15820^T^ and *L. chiayiensis* NCYUAS^T^ and 99.21% to *L. casei* ATCC 393^T^. This result indicated that analysis using only the 16S rRNA gene alone could not be used to identify the species of bacteria in this group, especially to distinguish between *Lacticaseibacillus* spp. and its subspecies. Recently, genome-based taxonomic approaches such as ANI (<95%) (Richter and Rosselló-Móra, 2009), AAI (<95–96%) (Konstantinidis et al., 2017), and dDDH (<70%) (Auch et al., 2010; Thompson et al., 2013) have been used as the thresholds recommended for differentiating strains into different species; the values in parentheses are cut-off values. The genomic attributes of strain TISTR 2593 are shown in Table 1.

**Table 1:** Genomic attributes of strain TISTR 2593.

Attributes	TISTR 2593
Accession no.	JBCAQN000000000
Genomic size (bp)	3,192,332^B^
Genome qualities	
Genome quality	Good^A^
CheckM completeness (%)	100^A^
Coarse consistency	99.9^A^
Fine consistency	98.4^A^
G + C content (%)	46.4^B^
N50	3,142,915^A^
L50	1^A^
No. of contig	6^B^
No. of subsystem	237^B^
No. of coding sequences	3,207^B^
No. of RNA	73^B^

^AData obtained from BV-BRC web-based tool; BData obtained from RAST sever web-based tool.^

On the basis of genomic analysis, strain TSITR-2593 was found to have an average average nucleotide number based on BLAST (ANIb) (97.65–77.45%), an average average nucleotide number based on MUMmer (ANIm) (98.54–84.48%), an average amino acid identity (AAI) (97.12–83.82%), and digital DNA–DNA hybridization (dDDH) (86.50–23.00%) (the ORGI values in Table 2) values that were significantly higher than the above cut-off values for species demarcation, indicating that the strain can be identified as *Lacticaseibacillus paracasei*. Precise taxonomic classification is of utmost importance when assessing potential risks associated with a particular taxon. By employing the protocol implemented in this study, the issue of misidentification or the inability to differentiate between closely related species, as observed in identification on the basis of biochemical assays and 16S rRNA gene analysis, can be mitigated.

**Table 2:** ANIb, ANIm, AAI and the digital DNA–DNA hybridization (dDDH) values between the genomes of strain TISTR 2593; *Lacticaseibacillus casei* ATCC 393^T^; *L. paracasei* subsp. *paracasei* ATCC 25302^T^; *L. paracasei* subsp. *tolerans* DSM 20258^T^; *L. zeae* DSM 20178^T^; and *L. chiayiensis* NCYUAS^T^.

Query genome	Reference genome	ANIb	ANIm	AAI	% dDDH (Formular 2*)	Model C.I. (%)	Distance	Prob. DDH > = 70%	G + C difference
1	2	97.65	98.54	96.65	86.50	83.9–88.7	0.0160	94.44	0.11
1	3	97.52	98.39	97.12	85.20	82.5–87.6	0.0174	93.83	0.01
1	4	78.52	85.80	84.38	25.20	22.9–27.7	0.1724	0.01	1.47
1	5	77.84	85.29	83.92	24.20	21.0–26.7	0.1800	0.01	1.33
1	6	77.45	84.48	83.82	23.00	20.7–25.5	0.1900	0.00	0.73

Genomic data: 1, strain TISTR 2593 (JBCAQN000000000); 2, *L. paracasei* subsp. *paracasei* ATCC 25302^T^ (ACGY00000000); and 3, *L. paracasei* subsp. *tolerans* DSM 20258^T^ (AYYJ00000000); 4, *L. casei* ATCC 393^T^ (AP012544.1-AP012546.1); 5, *L. zeae* DSM 20178^T^ (AZCT00000000); and 6, *L. chiayiensis* NCYUAS^T^ (MSSM00000000).

*Recommended formula (identities/HSP length), which is liberated of genome length and is thus prosperous against the use of complete/draft genome.

The historical use of bacteria within the genus *Lacticaseibacillus* (formerly known as *Lactobacillus*) in fermenting foods underscores the high number of generally recognized as safe (GRAS) species within this group (Salvetti et al., 2012). In recent decades, the global rise in obesity, which is linked to sedentary lifestyles and poor diets, has become a major health concern (Lin and Li, 2021). With some drugs used in clinical practice, such as orlistat and sibutramine, which have serious side effects, probiotic therapy has emerged as a safer alternative for treating obesity. Various experimental and clinical studies have highlighted the close relationships among probiotics, obesity, and the intestinal microbiome (Crovesy et al., 2020). The antiobesity effects of probiotics are attributed to their ability to modulate microbial communities, reduce lipid accumulation, and improve fat metabolism (Kobyliak et al., 2016; León Aguilera et al., 2022).

*L. paracasei* is widely acknowledged as one of the most commonly used probiotic bacteria in dairy and non-dairy products. Research has explored its health-promoting properties, including its ability to inhibit intestinal pathogens, modulate immune responses, improve lipid metabolism, and exert hypocholesterolemic and stress-reducing effects (Bengoa et al., 2021; Lee et al., 2023). In our study, we evaluated the hemolytic activity, antagonistic activity, antibiotic susceptibility, acid and bile salt tolerance, and adherence ability of *L. paracasei* TISTR 2593 to establish safety criteria and fundamental characteristics for probiotic bacteria.

### Hemolytic activity

*Lacticaseibacillus paracasei* TISTR 2593 exhibited non-hemolytic activity (*γ*-hemolysis) when cultivated on sheep blood agar. The absence of hemolytic activity is a fundamental criterion for selecting probiotic strains, ensuring the nonvirulent nature of the strain (Food, Agriculture Organization of the United, N., & World Health, O, 2006). This study aimed to evaluate the probiotic potential and antiadipogenic ability of the selected LAB strain; thus, we conducted tests on its probiotic properties and antiadipogenic activities.

The basic criteria for selecting probiotics include (1) being generally recognized as safe (GRAS); (2) demonstrating resistance to gastric conditions and bile salts; (3) adhering to the host’s intestinal epithelium; (4) exhibiting antagonistic activity against pathogenic bacteria; and (5) maintaining viability during processing and storage (Behnsen et al., 2013; Fijan, 2014; Rolfe, 2000). The absence of hemolytic activity and antibiotic resistance are pivotal characteristics of probiotics (Georgieva et al., 2015).

Hemolysis is considered a significant virulence factor among pathogenic microorganisms (Lahtinen et al., 2009). Our findings indicate that *L. paracasei* TISTR 2593 did not display hemolytic activity (γ-hemolysis). Several studies have documented the nonhemolytic nature of *L. paracasei*, suggesting the absence of hemotoxin production and the overall safety of these strains (Mangia et al., 2019; Tang et al., 2021; Xu et al., 2019).

### Antimicrobial activity

**Figure 1:**
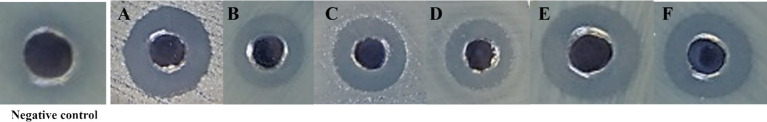
Antimicrobial activity of *L. paracasei* TISTR 2593 against pathogenic bacteria. **(A)** *Staphylococcus aureus* ATCC 6538, **(B)** *Escherichia coli* ATCC 8379, **(C)** *Salmonella* Typhimurium TISTR 292, **(D)** *Salmonella enteritidis* TISTR 15676, **(E)** *Listeria monocytogenes* DMST 13820, and **(F)** *Helicobacter pylori* PT14. Antimicrobial activity was assessed using the agar-well diffusion method, with sterile MRS broth as the negative control. Clear inhibition zones indicate bacterial susceptibility to *L. paracasei* TISTR 2593.

**Figure 2:**
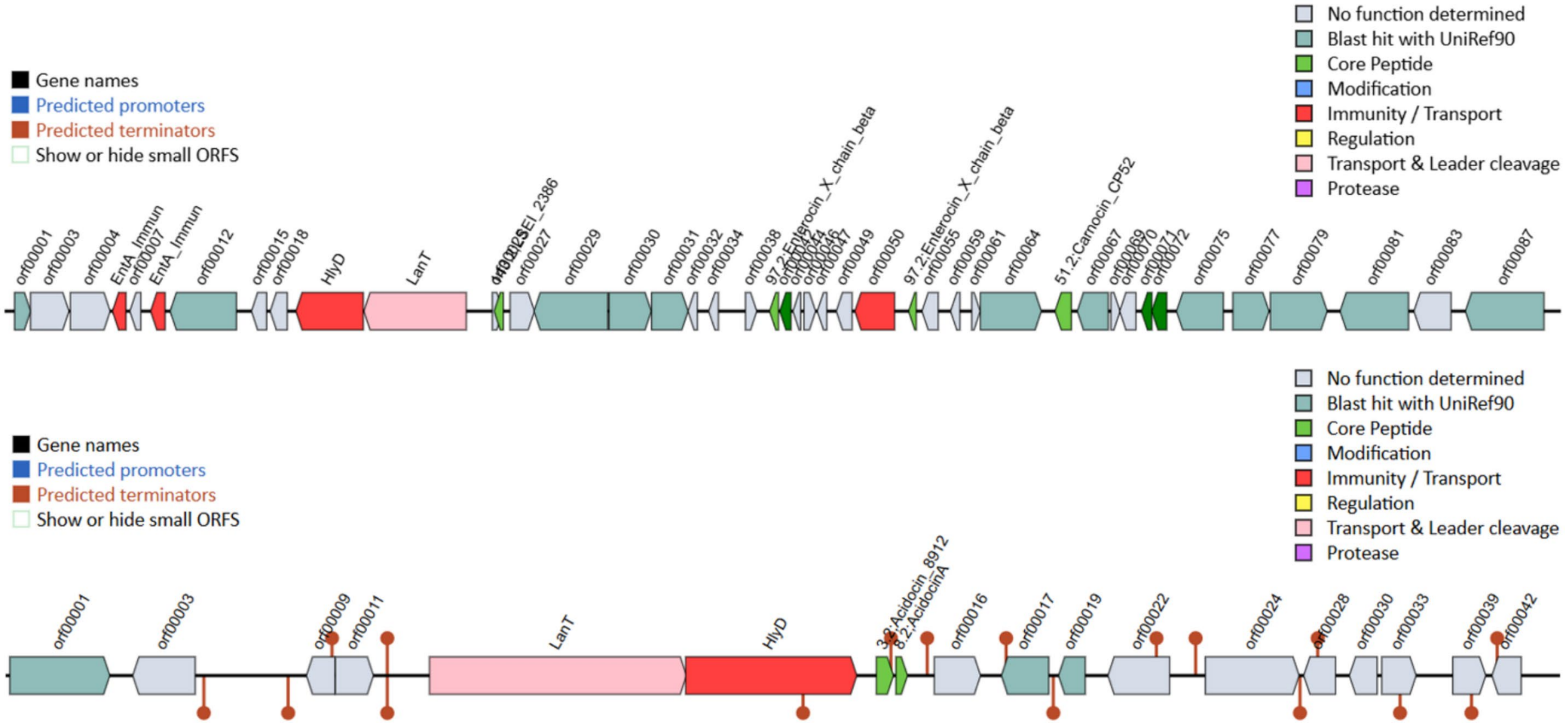
Bacteriocin-related genes were analyzed using the BAGEL4 program with the whole-genome sequence of strain TISTR 2593. This confirms the genetic potential for bacteriocin production, which may contribute to its antimicrobial activity.

*Lacticaseibacillus paracasei* TISTR 2593 exhibited potent antimicrobial activity against a range of pathogens, including *S. aureus* ATCC 6538, *E. coli* ATCC 8379, *Sal.* Typhimurium TISTR 292, *Li. monocytogenes* DMST 13820, and *H. pylori* PT14 (Table 3 and Figure 1). The majority of the inhibition zone diameters against these pathogenic bacteria were greater than 15 mm. The observed growth inhibition property of the isolate may be attributed to the production of inhibitory substances by *L. paracasei* TISTR 2593. Our findings on the adhesion ability of *L. paracasei* TISTR 2593 revealed a robust adherence rate to Caco-2 human epithelial cell lines. Furthermore, regarding antimicrobial activity, strain TISTR 2593 exhibited antagonistic activity against all six test pathogens, which are known human intestinal pathogens. LAB strains are known to produce various antimicrobial substances, including lactic acid, propionic acid, acetic acid, formic acid, fatty acids, hydrogen peroxide, and bacteriocins (De Vuyst and Leroy, 2007; Kingkaew et al., 2023b). Additionally, *in silico* BAGEL4 analysis revealed the presence of several bacteriocin-producing genes, such as Acidocin8912 (bit score = 97.0561), AcidocinA (bit score = 47.3654), CarnocinCP52 (bit score = 73.1738), and EnterocinX (bit score = 41.5874), as shown in Figure 2. Antoshina et al. (2022) reported that Acidocin could inhibit *S. aureus*, *Li. monocytogenes*, and *E. coli*. It may also have an impact on the digestive tract’s ability to function by directly inhibiting several symbiotic and pathogenic bacterial groups as well as through immunomodulatory actions. Furthermore, carnocin limited the growth of *Salmonella* sp. and *Staphylococcus* sp. (Maurya and Thakur, 2012). These findings suggest that *L. paracasei* TISTR 2593 has promising potential as a probiotic candidate capable of competing with and inhibiting the growth of pathogenic microbiota.

**Table 3:** Acid and bile salt tolerance, antibiotic susceptibility and Caco-2 cell adhesion ability of *L. paracasei* TISTR 2593.

% Survival ability		Caco-2 cell adhesion (%)	Antimicrobial activity (mm)					
Acid tolerance	Bile salt tolerance		*S. aureus* ATCC 6538	*E. coli* ATCC 8379	*Sal.*Typhimurium TISTR 292	*Sal. enteritidis* DMST 15676	*Li. monocytogenes* DMST 13820	*H. pylori* PT14
91.23 ± 2.64	88.72 ± 0.91	87.15 ± 0.84	+++	+++	+++	++	+++	+++

^a^ (−) no inhibition, (+) inhibition zone 0.1–7.0 mm; (++) inhibition zone 7.1–15.0 mm; (+++) inhibition zone > 15.0 mm.

### Antibiotic susceptibility

To ensure the safety of *L. paracasei* TISTR 2593, which lacks acquired antibiotic resistance and the ability to transfer antibiotic resistance genes to gut pathogens, we conducted an antibiotic sensitivity test via antibiotic disk agar diffusion. In this study, *L. paracasei* TISTR 2593 was susceptible to ampicillin, chloramphenicol, tetracycline, erythromycin, and clindamycin but resistant to vancomycin and kanamycin (Table 4). Resistance to vancomycin and kanamycin is common among *Lactobacillus* species. Vancomycin, a glycopeptide antibiotic, inhibits cell wall synthesis by targeting peptidoglycan synthesis. *Lactobacillus* species resist vancomycin because of the presence of D-Ala-D-Lac in the peptidoglycan instead of the dipeptide D-Ala-D-Ala, which prevents vancomycin binding. Kanamycin, an aminoglycoside antibiotic, inhibits protein synthesis and is intrinsically present in *Lactobacillus* owing to a lack of cytochrome-mediated electron transport, which mediates drug uptake. Since several *Lactobacillus* species are intrinsically resistant and nontransmissible, they are generally considered acceptable and do not pose major safety concerns. The results of hemolytic activity and antibiotic resistance in this study suggest that *L. paracasei* TISTR 2593 can be considered suitable for use as a probiotic.

**Table 4:** Antibiotic susceptibility of the strain TISTR 2593.

Antibiotics	*L. paracasei* TISTR2593
Ampicillin (10 μg)	S
Chloramphenicol (30 μg)	S
Clindamycin (2 μg)	S
Erythromycin (15 μg)	S
Kanamycin (30 μg)	R
Tetracycline (30 μg);	S
Vancomycin (30 μg)	R

Result Interpretation according to the CLSI breakpoints. R, resistant; S, sensitive.

### Acid and bile salt tolerance

*Lacticaseibacillus paracasei* TISTR 2593 exhibited a notably high survival rate after incubation under both acidic conditions and in a bile salt solution (Table 3). The survival rate was 91.23 ± 2.64% after a 3-h incubation in highly acidic conditions (pH 2.0) and 88.72 ± 0.91% after incubation in MRS broth containing 0.3% (w/v) bile salt (pH 8.0), indicating the probiotic potential of this LAB strain. Probiotics must survive gastrointestinal and bile conditions to maintain and exert their health-promoting effects. The findings of this study suggest that *L. paracasei* TISTR 2593 can indeed survive exposure to simulated gastric juice (pH 2) and bile acids (pH 8). This finding aligns with several reports indicating that *Lactobacillus* strains can tolerate simulated gastric conditions (pH 2.0–3.0) (Kim et al., 2021; Kingkaew et al., 2022a; Kingkaew et al., 2023a; Poornachandra Rao et al., 2015; Wang et al., 2018).

In response to acid stress, *Lactobacillus* species employ various mechanisms, including intracellular pH regulation, preservation of cell membrane functionality, and induction of stress response proteins (Wu et al., 2014). Bile salts are biological compounds capable of damaging DNA and membranes, leading to cell damage (Merritt and Donaldson, 2009). With respect to bile salt tolerance, several previous studies reported that many *Lactobacillus* species are resistant to bile salts (0.3–0.5% w/v), which is consistent with the findings of the present study (Kingkaew et al., 2022a; Kingkaew et al., 2023a; Kingkaew et al., 2022b). *Lactobacillus* and *Bifidobacterium* possess numerous proteins dedicated to effluxing bile salts or protons, modifying sugar metabolism, or preventing protein misfolding in response to bile salt stress (Ruiz and Margolles, 2013).

### Adhesion assay

The Caco-2 cell serves as a widely accepted *in vitro* system for assessing the adhesion capabilities of potential probiotics. *L. paracasei* TISTR 2593 demonstrated an adhesion rate of 87.15 ± 0.84% (Table 3), indicating its ability to adhere, establish, and colonize the gastrointestinal tract, thereby increasing its potential for survival. In addition to surviving the harsh conditions of the stomach and intestinal tract, effective probiotics must also attach to intestinal epithelial cells to colonize and defend against pathogens (Kingkaew et al., 2022a; Kingkaew et al., 2023a; Kingkaew et al., 2022b; Kingkaew et al., 2023b). On the basis of genomic research, genes that encode moonlighting proteins, such as elongation factor Tu and the chaperonin *GroEL*, display multifunctionality and are associated with adhering to epithelial cells (Kingkaew et al., 2022b). Therefore, these proteins can support adhesion ability.

### Cholesterol assimilation assay

To further evaluate the probiotic performance, we analyzed the cholesterol-lowering capacity of *L. paracasei* TISTR 2593. Dyslipidemia is a modifiable risk factor for cardiovascular disease (CVD), a leading cause of mortality (Kingkaew et al., 2022a). Hence, reducing cholesterol levels is vital for prevention. *In vitro* cholesterol assimilation tests revealed that the TISTR 2593 strain demonstrated a potential 69.48 ± 0.94% reduction in cholesterol concentration. Strain TISTR 2593 has the capacity to absorb cholesterol, which lowers the quantities of luminal cholesterol that are available for absorption (Kingkaew et al., 2022a; Kingkaew et al., 2023a; Kingkaew et al., 2022b; Kingkaew et al., 2023b; Tarrah et al., 2021).

### Cytotoxicity

**Figure 3:**
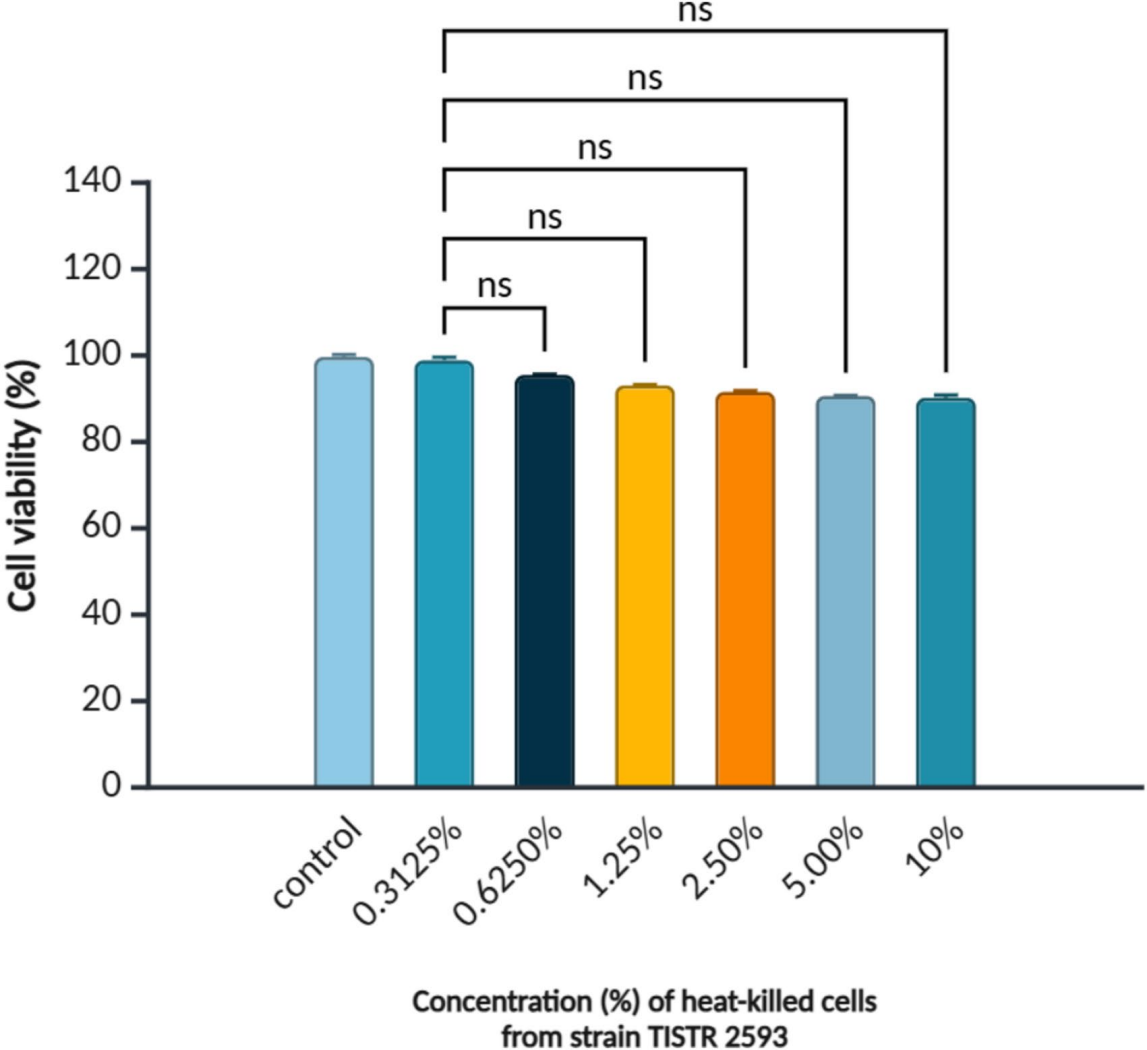
Viability of 3T3-L1 pre-adipocytes treated with *L. paracasei* TISTR 2593. Cell viability was assessed using the MTT assay after treatment with different concentrations (0.3125–10.00%) of heat-inactivated *L. paracasei* TISTR 2593. Data are presented as mean ± SD (*n* = 3). Statistical significance is indicated as *p < 0.05; ns = no significance.*

The cytotoxicity of heat-inactivated concentrations of the strain TISTR 2539, ranging from 0.3125 to 10% (v/v), was tested in pre-confluent 3T3-L1 cells. Exposure to heat-inactivated TISTR 2593 cells for 48 h resulted in a reduction in cell viability of up to 85% (Figure 3). Since no significant difference was observed between the lowest and highest tested concentrations (0.3125 and 10% v/v), concentrations within this range were selected for lipid accumulation and adipogenic gene expression analysis.

Given the research focus on the antiadipogenic potential of strain TISTR 2593, further examination of its cytotoxicity and ability to reduce lipid accumulation was conducted using 3T3-L1 mouse preadipocyte cells. *L. paracasei* TISTR 2593 significantly decreased lipid droplet accumulation in 3T3-L1 adipocytes without exhibiting cytotoxic effects. Treatment with 10% (v/v) heat-inactivated *L. paracasei* TISTR 2593 cells resulted in an approximately 50% reduction in lipid accumulation. Numerous studies have reported that *Lactobacillus* strains can diminish lipid accumulation by inhibiting adipocyte differentiation (Huang et al., 2019; Kim et al., 2021).

### Lipid accumulation

**Figure 4:**
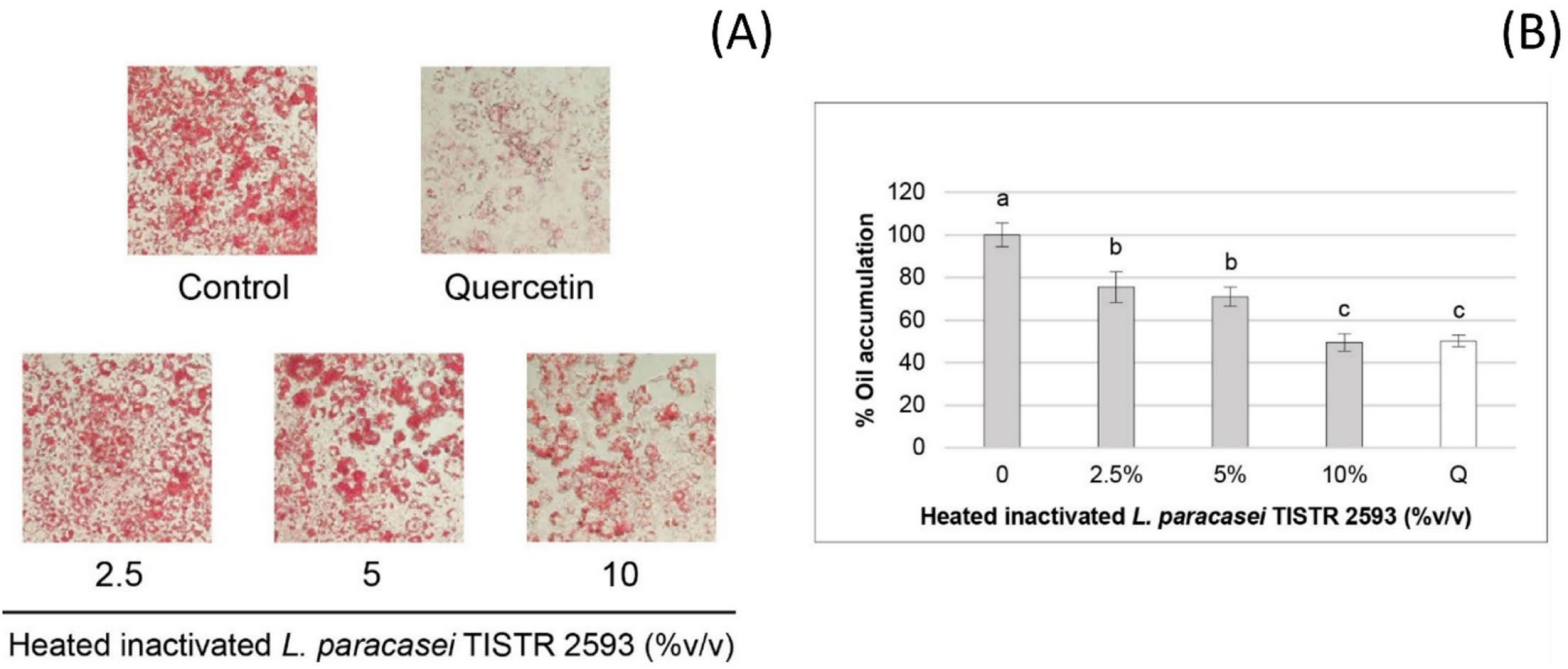
Lipid accumulation in 3T3-L1 pre-adipocytes treated with *L. paracasei* TISTR 2593. **(A)** Lipid droplet formation was observed in pre-adipocytes differentiated with and without *L. paracasei* TISTR 2593 at various concentrations. **(B)** The percentage of lipid accumulation was quantified relative to the control. Data are expressed as mean ± SD from three independent experiments. Different letters (a–c) indicate significant differences (*p <* 0.05).

The quantitative analysis of Oil-Red-O staining (Figure 4A) was conducted to assess lipid accumulation, which is presented as a percentage (Figure 4B). The results clearly demonstrated that *L. paracasei* TISTR 2593 significantly reduced lipid droplet accumulation in 3 T3-L1 adipocytes in a dose-dependent manner. At a concentration of 10% (v/v) of the strain TISTR 2593, the reduction in lipid accumulation mirrors that of the positive control (quercetin), showing a 50% reduction (Figure 4B).

Additionally, *L. paracasei* TISTR 2593 exhibited a notable 69.48 ± 0.94% assimilation of cholesterol. *Lactobacillus* strains have been reported to employ various mechanisms for cholesterol removal, including bile-salt hydrolase (BSH) activity, production of short-chain fatty acids (SCFAs), cholesterol conversion into coprostanol, and cholesterol adsorption onto the cell wall, leading to assimilation into the cell membrane (Brashears et al., 1998; Gilliland et al., 1985; Lye et al., 2010; Smet et al., 1994).

These findings suggest that *L. paracasei* TISTR 2593 possesses promising probiotic properties and anti-obesity effects. However, further investigations are necessary to validate its efficacy as an anti-obesity probiotic through *in vivo* studies using a high-fat diet-induced obese mouse model.

### Expression of the C/EBP-*α* and PPAR-*γ* genes

**Figure 5:**
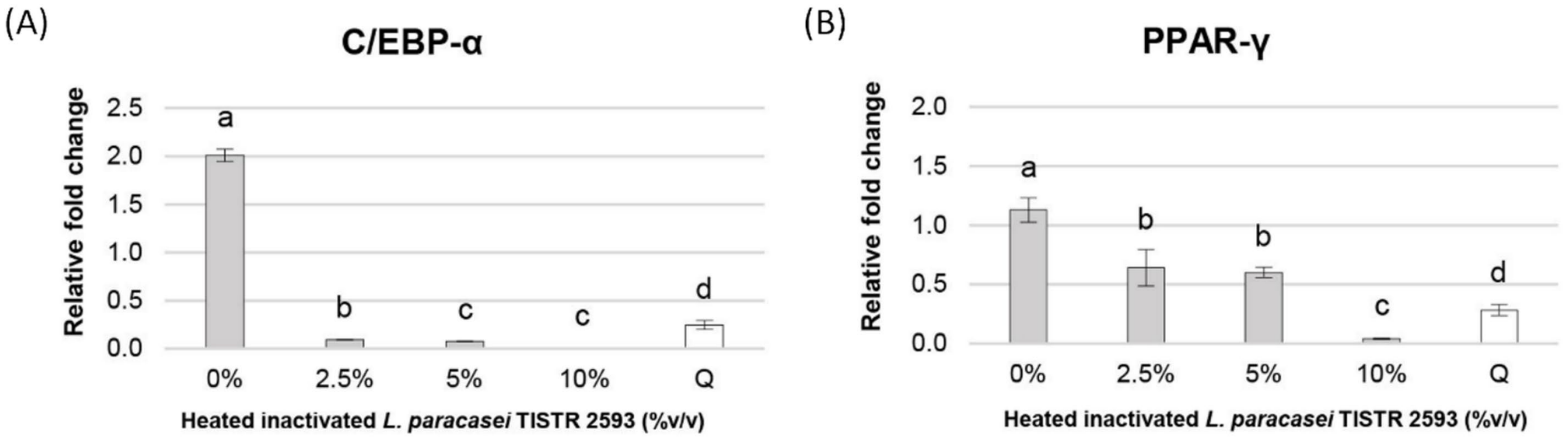
Expression of PPAR-*γ* and C/EBP-*α* genes in 3T3-L1 pre-adipocytes. Gene expression of PPAR-γ and C/EBP-α was analyzed after treatment with different concentrations of heat-inactivated *L. paracasei* TISTR 2593. Data are expressed as mean ± SD from three independent experiments. Different letters (a–c) indicate significant differences (*p < 0.05*).

Many studies have shed light on the role of the CCAAT-enhancer-binding protein-α (C/EBP-α) and peroxisome proliferator-activated receptor-γ (PPAR-γ) genes in the molecular regulation of adipocyte differentiation or adipogenesis (Kim et al., 2007). Our findings revealed that all concentrations of heat-inactivated *L. paracasei* TISTR 2593 at 2.5, 5, and 10% (v/v) were capable of reducing the expression of both the PPAR-γ and C/EBP-α genes, with particularly pronounced suppression observed at 10% (v/v), nearly reaching 100% (Figure 5). These results suggest a decrease in lipid droplet formation in 3T3-L1 adipocytes following treatment with heat-inactivated *L. paracasei* TISTR 2593 cells, which is indicative of a disturbance in adipogenesis.

To validate the antiadipogenic effect of *L. paracasei* TISTR 2593, we examined the expression of adipogenesis-related genes via qRT–PCR. Previous studies have underscored the functional importance of the PPAR-γ and C/EBP-α genes in the molecular regulation of the adipocyte differentiation process (Bergen and Burnett, 2013; Farmer, 2006). This process involves the conversion of preadipocytes into mature adipocytes, in which PPAR-γ and C/EBP-α serve as crucial transcription factors that act as central positive regulators of adipogenesis (Khalilpourfarshbafi et al., 2019). A reduction in lipid droplet formation is correlated with decreased expression of the PPAR-γ and C/EBP-α genes, indicating a disruption in adipogenesis (Ali et al., 2013; Rosen and MacDougald, 2006), which is consistent with our observations.

Treatment with *L. paracasei* TISTR 2593 led to a dose-dependent decrease in both PPAR-γ and C/EBP-α expression levels, accompanied by a reduction in lipid droplet formation. Notably, at a concentration of 10% (v/v) *L. paracasei* TISTR 2593, the expression levels of these adipogenic markers were significantly downregulated, nearly reaching 100%, whereas lipid accumulation decreased by approximately 50%. These findings suggest that *L. paracasei* TISTR 2593 suppresses lipid accumulation during adipocyte differentiation by downregulating lipogenic factors.

### *In silico* safety assessment

**Figure 6:**
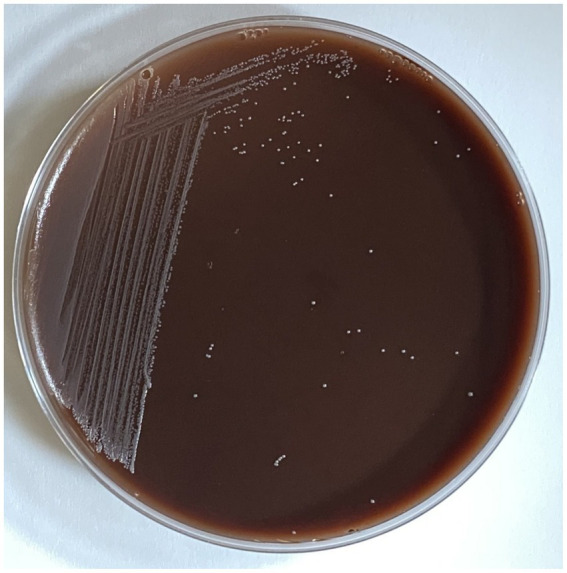
Hemolytic activity of *L. paracasei* TISTR 2593 on sheep blood agar. Hemolysis was assessed by streaking *L. paracasei* TISTR 2593 on sheep blood agar. No hemolytic activity was observed, indicating the strain’s potential safety for further applications.

The genomic analysis of strain TISTR 2593 provides compelling evidence supporting its potential as a safe strain, as summarized in Table 5. When strains are selected for probiotic applications, it is imperative to conduct thorough safety assessments and scrutinize their genetic composition for potential virulence, pathogenicity, or toxicity factors. The genome of strain TISTR 2593 strongly supports its probiotic potential. PathogenFinder predictions indicate that TISTR 2593 is anticipated to be a nonhuman pathogen, reinforcing its suitability for probiotic use. The VirulenceFinder tool was also employed for genome analysis, revealing the absence of recognized virulence determinants or genes. Notably, the hemolysis III (*hlyIII*) gene was identified in the TISTR 2593 genome. While this gene is not exclusive to the strain and has been found in various commercial probiotics, such as *L. plantarum* 299V and *L. rhamnosus* GG (Kingkaew et al., 2023b), both of which are recognized as safe for consumption, strain TISTR 2593 exhibited gamma hemolytic activity (nonhemolysis) on sheep blood agar (Figure 6). Without other pathogenesis genes in the genome, the presence of the hemolysis III gene does not pose a safety risk. Additionally, these virulence genes may confer advantages to the bacterium by enhancing its endurance, which can be beneficial in conditions requiring viable bacteria, such as probiotics and starters (Kingkaew et al., 2022b).

**Table 5:** Pathogenicity prediction, prophage region detection and antimicrobial resistance (AMR) analysis of strain TISTR 2593.

Attribute/Strain	TISTR 2593	*L. plantarum* 299V	*L. rhamnosus* GG
Probability of being a human^a^ pathogen	0.189	0.185	0.198
Input proteome coverage (%)^a^	16.1	0.48	40.5
Matched pathogenic families^a^	0	0	0
Matched not pathogenic families^a^	492	15	1,147
Conclusion^a^	Non-human pathogen	Non-human pathogen	Non-human pathogen
No. of plasmid(s)^b^	0	2 (rep28, rep38)	0
No. of phage region(s)^c^	7	4	5
AMR genes:			
CARD^d^:			
- No. of perfect hits	0	0	0
- No. of strict hits	1 (*qacJ*)	0	0
- No. of loose hits	211	194	207
ResFinder^e^	No resistance	No resistance	No resistance

^**aData obtained from PathogenFinder; bData obtained from PlasmidFinder; cData obtained from PHASTER; and eData obtained from ResFinder.**^

Evaluating biogenic amine (BA) production also has significant implications. Following the guidelines issued by the European Food Safety Authority (EFSA), the adoption of BA-nonproducing strains as a precautionary measure to mitigate the potential risks associated with BAs is recommended (Kingkaew et al., 2023b). To ascertain the BA-nonproducing nature and safety of a given strain in this context, it is possible to investigate the associated BA genes. According to the KEGG annotation, strain TISTR 2593 lacks genes related to the production of biogenic amines (BAs), including tryptamine, tyramine, histamine, ornithine, spermine, spermidine, and cadaverine, within its genome. However, the ornithine decarboxylase gene was detected in the strain TISTR 2593. Importantly, the presence of ornithine decarboxylase genes does not pose a safety risk, but it is linked to acid stress mechanisms, which modulate the intracellular pH and support the viability of probiotics under gastrointestinal conditions, as proposed by Ferreira et al. (2015). Consequently, strain TISTR 2593 can be categorized as a BA nonproducer, posing no safety concerns.

Additionally, the genome of strain stain TISTR 2593 was annotated with the D-lactate dehydrogenase (D-LDH) gene. The presence of D-lactate dehydrogenase (D-LDH) proves advantageous, rendering it suitable for starter and bioplastic applications. D-lactate production can potentially lead to D-lactate acidosis.

A comprehensive investigation revealed that strain TISTR 2593 presents unique and distinctive features for probiotic applications. Its genome underscores its safety, as it lacks virulence factors and antimicrobial resistance genes. It is stress resistant and pH stable and does not produce biogenic amines. Moreover, its genetic makeup is well suited for probiotic and starter applications. These characteristics position TISTR 2593 as a promising and original probiotic candidate, warranting further research to fully explore its potential.

To identify ARG genes and mobile genetic elements, whole-genome sequencing (WGS) has proven to be a robust tool for addressing concerns related to antibiotic resistance genes and virulence factors. This study utilized genomic methodologies, contributing significantly to our understanding of the probiotic and biotechnological capabilities inherent in strain TISTR 2593.

A comprehensive analysis revealed the absence of ARG genes in the Resfinder 4.4.1 database. In contrast, the CARD database search, which employs default parameters (perfect and strict hits only), identified one gene, *qacJ* (a small multidrug resistance (SMR) antibiotic efflux pump). However, adjusting the parameters to include perfect, strict, and loose hits resulted in 212 hits (0 perfect hits, one strict hit, and 211 loose hits), with an identity range of 20.57–72.19% for the matching regions. The strict and loose hits encompassed genes associated with various antibiotic resistance mechanisms, including antibiotic target alteration (52), reducing permeability to antibiotics (1), antibiotic target protection (11), antibiotic efflux (140), antibiotic inactivation (7), and antibiotic target replacement (4).

While the Resfinder and CARD databases focus primarily on antimicrobial resistance genes in pathogenic bacteria, potentially overlooking those in nonpathogenic bacteria, our exploration of the KEGG database revealed two AMR-related genes in the TISTR 2593 genome (Table 6). Notably, strain TISTR 2593 contained one beta-lactamase gene, and the strain was sensitive to ampicillin. Given the diversity within the beta-lactamase enzyme family and variations in substrate specificity (Philippon et al., 2016), resistance to other beta-lactam drugs cannot be ruled out without further investigation. Moreover, multidrug resistance (*abcA* and *bmrA*) has been detected in several probiotic strains (*L. plantarum* 299V, *L. plantarum* WCFS1, and *L. plantarum* JDM1) (Chokesajjawatee et al., 2020).

**Table 6:** List of antimicrobial resistance genes and their locations in TISTR 2593 genome.

Resistance	KEGG_ID	Gene Name
Beta-lactam resistance	K17836	*penP* beta-lactamase class A [EC:3.5.2.6]
Multidrug resistance	K18104	*abcA*, *bmrA* ATP-binding cassette, subfamily B, bacterial *AbcA/BmrA* [EC:7.6.2.2]

In this study, data was obtained from KEGG database.

The findings from the *oriT*Finder and PlasmidFinder tools revealed that strain TISTR 2593 lacks both plasmids and *oriT*, indicating its inability to self-transmit through conjugative transfer. Additionally, no antibiotic resistance genes (ARGs) were identified in the prophage regions. These results collectively establish that the absence of ARGs in TISTR 2593 eliminates the risk of their transfer to other bacteria. The presence of two intact, four questionable, and two incomplete prophage regions in the genome is intriguing, suggesting the potential role of these regions in facilitating strain adaptation and survival in harsh environments, as indicated by Phuengjayaem et al. (2023). Moreover, the incorporation of two phage lysins within the prophage region of the genome is particularly noteworthy. These lysins, known for their ability to inhibit pathogens (Phuengjayaem et al., 2023), further accentuate the promise of strain TISTR 2593 as a safe and effective probiotic. Consequently, the strain poses no safety concerns regarding functional and transferrable ARG characteristics (Kingkaew et al., 2022b). Therefore, strain TISTR 2593 is deemed safe and is considered a nonantibiotic source.

## Conclusion

In conclusion, the isolation and characterization of the strain TISTR 2593, *Lacticaseibacillus paracasei,* underscore its potential as a safe and effective probiotic candidate. Through genomic analysis and phenotypic assays, we elucidated its nonhemolytic nature, potent antimicrobial activity, susceptibility to antibiotics, and robust survivability under acidic and bile salt conditions. Furthermore, its ability to adhere to intestinal cells, assimilate cholesterol, and exert antiadipogenic effects without causing cytotoxicity highlights its promising probiotic properties. Importantly, genomic scrutiny revealed the absence of virulence factors and antimicrobial resistance genes, ensuring its safety for consumption. These findings position strain TISTR 2593 as a noteworthy contender for further exploration in probiotic applications, warranting future *in vivo* studies to validate its health-promoting effects. This comprehensive investigation contributes valuable insights into the probiotic potential of *L. paracasei* TISTR 2593 and underscores its importance in promoting gastrointestinal health and combating obesity-related concerns.

## Data Availability

The datasets presented in this study can be found in online repositories. The names of the repository/repositories and accession number(s) can be found in the article/supplementary material. This whole genome project has been deposited in the NCBI under the accession number: JBCAQN000000000.
